# Reclaiming momentum: The Essential Programme on Immunization in the journey to 2030 and beyond

**DOI:** 10.1080/21645515.2025.2580134

**Published:** 2025-10-27

**Authors:** Charles S. Wiysonge, Sara Cooper, Chinwe J. Iwu-Jaja, Abdu A. Adamu, Miluka P. Gunaratna, Balcha G. Masresha

**Affiliations:** a Cochrane South Africa, South African Medical Research Council, Cape Town, South Africa; b Centre for Evidence-Based Health Care, Division of Epidemiology and Biostatistics, Department of Global Health, Stellenbosch University, Cape Town, South Africa; c Division of Social and Behavioural Sciences, School of Public Health and Family Medicine, University of Cape Town, Cape Town, South Africa; d Vaccine-Preventable Diseases Programme, World Health Organization Regional Office for Africa, Brazzaville, Congo

**Keywords:** Immunization Agenda 2030, vaccine hesitancy, equity, financing, implementation research

## Abstract

Fifty-one years after the launch of the Expanded Programme on Immunization – now rebranded as the Essential Programme on Immunization – routine vaccination remains the most transformative public health intervention, preventing more than 154 million deaths globally. Yet despite this historic gain, the world is at a critical juncture. Since 2020, more than 20 million children are left under-vaccinated each year, leading to outbreaks of vaccine-preventable diseases. In our view, the greatest risk is the normalization of stagnation, where failure to attain optimal coverage is passively accepted as the new normal. This moment demands more than recovery; it requires renewed momentum, bold accountability, and strategies tailored to local realities. We argue that three accelerators are essential: embedding immunization within resilient primary health care systems, addressing vaccine hesitancy through trust-building and continuous engagement, and securing sustainable financing linked to equity goals. Implementation science must inform these actions, ensuring lessons are documented and adapted across contexts. The next 5 y, leading to 2030, will decide whether immunization delivers on its promise of protection for all or retreats into entrenched inequities. We contend that only political will, solidarity, and systematic learning can safeguard vaccines as a global public good for future generations.

Vaccination is the most transformative public health intervention of the past half-century, yet its continued impact is at risk if the global community normalizes stagnation.^1–6^ Since its establishment in 1974 by the World Health Organization (WHO), the Expanded Programme on Immunization – now rebranded as the Essential Programme on Immunization (EPI) – has played a central role in extending the benefits of vaccines worldwide.^2,3^ Routine childhood vaccination has prevented over 154 million deaths over the past five decades.^4^ At global level, vaccination coverage has expanded from 5% of the children receiving three doses of diphtheria-tetanus-pertussis containing vaccines (DTP3) in 1974 to 85% in 2024.^1,5,6^ In addition, the number of diseases with global recommendations for vaccination doubled from 7 before 1980 to 14 in 2024.^3^

This expansion has contributed not only to improved child survival but also to broader health and development outcomes. For every life saved, an average of 66 y of full health was gained, amounting to more than 10 billion healthy life years worldwide since 1974.^4^ Vaccination is credited as the single largest contributor to improved child survival over the past half-century.^4^ Protection now spans beyond childhood, with new vaccines such as human papillomavirus (HPV) and respiratory syncytial virus (RSV) signaling a life-course approach to vaccination.^3^ Vaccines also contribute to antimicrobial resistance mitigation by reducing infections requiring antibiotics.^7^ Yet, the durability of these gains depends on sustaining financing and integrating innovations – such as digital tools, artificial intelligence, and resilient supply chains – into everyday practice.

Despite this success, recent analyses show worrying trends.^8^ Global vaccination coverage gains have slowed or reversed in some countries and regions, particularly after the COVID-19 pandemic, which disrupted routine services and exacerbated inequities.^5,6^ The COVID-19 pandemic led to the largest backslide in three decades; for example, global DTP3 coverage fell from 86% in 2019 to 82% in 2021.^6^ Although there has been partial recovery since 2022, global DTP3 coverage remained below the pre-pandemic level at 85% in 2024, far short of the 90% Immunization Agenda 2030 (IA2030) target.^6,8^ Among the 2024 birth cohort, there were 19.9 million under-vaccinated children – including 14.3 million zero-dose children who had not received even a first dose of DTP and 5.7 million children who started but had not completed the series of three DTP doses.^6^ The distribution of missed children is highly inequitable. More than half of all zero-dose children in 2024 lived in just eight countries, namely, Nigeria, India, Sudan, Democratic Republic of Congo, Ethiopia, Indonesia, Yemen, and Afghanistan.^6^ In addition, within countries, subnational disparities are stark.^5^ These inequities are not random. They reflect conflict, fragility, systemic exclusion, and political neglect, underscoring the need for tailored strategies in immunization programs.^9^

Looking ahead to 2030, forecasts suggest that without substantially accelerated progress beyond current trends, many countries will not meet the IA2030 goals.^5,8^ The trajectory also highlights a widening gap between high-performing and lagging countries,^5^ demanding peer accountability frameworks and targeted solidarity to close disparities.^10^ These vaccination coverage gaps and inequities increase vulnerability to outbreaks. The number of large or disruptive outbreaks of vaccine-preventable diseases (VPDs) increased by 43% from 76 in 2019 to 109 in 2023 and remained at the same level in 2024.^8^ These include wild poliovirus with two large or disruptive outbreaks in 2019, two in 2023, and two in 2024; circulating vaccine-derived polioviruses with 22 in 2019, 34 in 2023, and 35 in 2024; cholera with one in 2019, seven in 2023, and six in 2024; and meningococcus with two in 2019, seven in 2023, and six in 2024. The most prevalent VPD was measles with 44 large or disruptive outbreaks reported in 2019, 57 in 2023, and 58 in 2024.^8^ The resurgence of VPD outbreaks underscores the urgent need to reinforce foundational immunization functions as a prerequisite for sustainable disease control. Accepting stalled coverage recovery as the new normal would be a grave mistake. Moving forward, progress depends on establishing robust peer accountability and designing tailored strategies to both increase routine vaccination coverage and manage outbreaks effectively.

While access barriers remain critical, vaccine hesitancy has gained prominence as a key driver of stagnating coverage.^11^ A decade of qualitative evidence reveals that vaccine hesitancy is not a monolithic phenomenon, but a dynamic, context-dependent process influenced by multiple socio-political factors.^12^ The first insight from the comprehensive qualitative evidence base is that vaccine hesitancy is not simply a product of misinformation or lack of awareness; in some cases, higher biomedical knowledge correlates with hesitancy. Secondly, experiences of marginalization and consequent distrust in institutions often fuel vaccine hesitancy, with vaccination refusals sometimes expressing broader political grievances. Thirdly, concerns differ across settings, vaccines, and populations – for instance, HPV vaccine hesitancy is often linked to sexuality norms, while COVID-19 vaccine hesitancy was tied to perceptions of novelty and speed. In addition, barriers in vaccine access and delivery can stimulate hesitancy due to the time, effort, and opportunity costs that accessing vaccination involves. Finally, caregiver vaccination decisions are fluid and subject to change over time, underscoring the importance of continuous engagement rather than one-off demand generation and communication campaigns.^12^ This dynamic nature makes it critical to embed continuous listening and community engagement within health systems, rather than treating demand generation as an external add-on.

Tackling vaccine hesitancy requires moving beyond risk communication and one-size-fits-all strategies. More multi-faceted and complex strategies are imperative if our interventions are to succeed.^13^ This includes addressing context-specific socio-behavioral and political drivers of vaccine hesitancy. It necessitates addressing the structural and service-level factors that hinder access and convenience around vaccination. It also involves strengthening community engagement to build institutional trust. In addition, it requires fostering local ownership and bottom-up approaches, empowering communities and local leaders as primary partners in vaccination initiatives. Africa’s rich history of community engagement and mobilization against poliomyelitis, HIV/AIDS, and Ebola demonstrates the power of community-led solutions.^14^ Building trust and resilience in vaccination demand clearly requires multidisciplinary research and insights from diverse intellectual fields, including those outside the traditional scope of vaccination programs.^15^ For example, socio-behavioral research is essential for developing tailored strategies that appropriately target the complex processes driving vaccine hesitancy in certain contexts.^16^ Emerging strategies must be evaluated through implementation research, capturing lessons that can be applied and adapted across diverse contexts.^17^

When communities lose trust in the healthcare system following an adverse event following immunization (AEFI), restoring confidence requires a thoughtful, inclusive, and well-coordinated response. Central to this effort is the strengthening of the pharmacovigilance system, which ensures rigorous monitoring of vaccine safety with timely and transparent responses to AEFIs. By reinforcing accountability and responsiveness, such systems help rebuild public trust and support the integrity of immunization programs. Addressing vaccine hesitancy is not merely a matter of improving confidence and uptake for routine vaccination, it is also a foundational requirement for pandemic preparedness. Without public confidence in vaccines and the systems that deliver them, efforts to rapidly deploy immunization strategies during future health emergencies will be severely compromised; undermining both national and global response capacities to contain outbreaks and protect vulnerable populations.

The IA2030 framework emphasizes that immunization must be embedded within strong primary health care (PHC) systems.^18^ This integration offers several benefits: efficiency and resilience; equity; and sustainability. In many countries, immunization platforms have always served as entry points for other essential services; from maternal health to infectious disease surveillance. Strengthening PHC can help reduce geographical and socio-economical barriers to access, reaching marginalized zero-dose populations.^19^ Such integration and strengthening can simultaneously address vaccine hesitancy that emerges from an exclusionary focus on vaccination or inequities in basic healthcare services.^13^ Embedding immunization financing within broader health budgets should also reduce reliance on external funding and improve program resilience. Modeling confirms that immunization remains one of the most cost-effective interventions, with returns on investment up to 52 times program costs.^20^ This high payoff underlines why sustaining and expanding immunization programs is so critical. Integration must also facilitate life-course vaccination, including adolescents and adults.^21^ In practice, this requires aligning immunization with broader PHC reforms and universal health coverage pathways to ensure sustainability.

Expanding to a life-course approach is essential for the next phase of the EPI. Historically centered on childhood, immunization must now protect individuals at every stage of life – infancy, adolescence, adulthood, pregnancy, and older age – to address shifting epidemiological and demographical realities. The Immunization Agenda 2030 recognizes life-course vaccination as a cornerstone of resilient and people-centered systems.^18,21^ Adolescent vaccination – through HPV vaccination and tetanus-diphtheria boosters – anchors engagement with schools and youth services; maternal immunization with tetanus, influenza, pertussis, and RSV protects mothers and newborns; and adult vaccination for influenza, hepatitis B, pneumococcal disease, and COVID-19 safeguards essential workers and individuals with chronic conditions. For older adults, vaccines against influenza, pneumococcal disease, and herpes zoster support healthy aging and reduce hospitalization. The global analysis published by Vilajeliu and colleagues in 2025 shows that although 90% of countries vaccinate adults against COVID-19, only 8% of low-income countries vaccinate older adults against influenza versus 89% of high-income countries – underscoring persistent inequities.^22^ Integrating vaccination opportunities across the life span within PHC, antenatal, occupational, and geriatric services can strengthen continuity of protection and pandemic preparedness. A true life-course approach transforms immunization from a child-focused program into a lifelong investment in health, equity, and resilience.

In recent years, there have been changes to the global health architecture and financing mechanisms, which can pose significant challenges in the short term to countries which have hitherto depended on external donor funding.^23^ However, this also heralds an opportunity for countries to reexamine their priorities and allocate resources toward self- sufficiency in funding key public health program areas like immunization.

The next 5 y, leading up to the IA2030 deadline, represent a critical window for course correction. Several opportunities stand out: catch-up and recovery initiatives such as the Big Catch-Up launched in 2023;^24^ life-course approaches including through HPV, RSV, and other new vaccines;^3,21^ and harnessing innovation in digital monitoring, artificial intelligence, community-based delivery models, and locally tailored communication strategies embedded with rigorous implementation research to foster equity.^9,13^ The expanding use cases and increasing importance of artificial intelligence tools should also be harnessed by national EPI programs to support data analytics and modeling, conduct better outbreak risk assessment, improve social listening, enhance vaccine logistics systems, and plan high quality and targeted interventions. In addition, peer accountability – where countries review one another’s progress – can provide the transparency and solidarity needed to maintain momentum. Regional peer learning and robust accountability frameworks will be indispensable for building strong immunization programs within the IA2030 framework. In the previous decade, the framework for monitoring the Addis Declaration on Immunization [ADI], which was signed by African Union Heads of State in 2017, served as a platform for tracking accountability toward the ADI goals on the continent.^25^

The 50th anniversary of EPI is both a celebration and a warning. Vaccination has saved more lives than any other health intervention in the past half-century; yet millions remain unprotected, inequities are widening, and VPDs are resurging. If the world accepts stalled coverage as the new normal, decades of progress risk being reversed. Achieving the IA2030 vision requires a dual focus: sustaining and financing strong immunization systems within PHC as well as addressing the complex social and political dynamics of vaccine hesitancy. Mid-way through the IA2030 decade, currently available data make clear that the world is off track, and the next 5 y will determine whether immunization continues to deliver transformative health gains or whether stagnation becomes entrenched. Safeguarding the future of immunization requires bold political will, systematic learning, and solidarity across geographies. Implementation research must guide context-sensitive strategies, while equity must remain the central measure of progress. We contend that the years 2026 to 2030 will determine whether immunization continues to deliver transformative health gains or whether inequities and vaccine hesitancy erode decades of progress. The journey to 2030 and beyond demands courage, accountability, and commitment to vaccination as a shared global good.
